# Seasonal and Geographical Variation of Dengue Vectors in Narathiwat, South Thailand

**DOI:** 10.1155/2016/8062360

**Published:** 2016-06-29

**Authors:** Ornanong Boonklong, Adisak Bhumiratana

**Affiliations:** ^1^Department of Mathematics and Statistics, Faculty of Science and Technology, Nakhon Si Thammarat Rajabhat University, Muang, Nakhon Si Thammarat 80280, Thailand; ^2^Center of Ecohealth Education and Research (CEER), Faculty of Public Health, Thammasat University, Rangsit Campus, Pathumthani 12121, Thailand

## Abstract

Using GIS-based land use map for the urban-rural division (the relative ratio of population density adjusted to relatively* Aedes*-infested land area), we demonstrated significant independent observations of seasonal and geographical variation of* Aedes aegypti* and* Aedes albopictus* vectors between Muang Narathiwat district (urban setting) and neighbor districts (rural setting) of Narathiwat, Southern Thailand, based on binomial distribution of* Aedes* vectors in water-holding containers (water storage containers, discarded receptacles, miscellaneous containers, and natural containers). The distribution of* Aedes* vectors was influenced seasonally by breeding outdoors rather than indoors in all 4 containers. Accordingly, both urban and rural settings elicited significantly seasonal (wet versus dry) distributions of* Ae. aegypti* larvae observed in water storage containers (*P* = 0.001 and *P* = 0.002) and natural containers (*P* = 0.016 and *P* = 0.015), whereas, in rural setting, the significant difference was observed in discarded receptacles (*P* = 0.028) and miscellaneous containers (*P* < 0.001). Seasonal distribution of* Ae. albopictus* larvae in any containers in urban setting was not remarkably noticed, whereas, in rural setting, the significant difference was observed in water storage containers (*P* = 0.007) and discarded receptacles (*P* < 0.001). Moreover, the distributions of percentages of container index for* Aedes*-infested households in dry season were significantly lower than that in other wet seasons, *P* = 0.034 for urban setting and *P* = 0.001 for rural setting. Findings suggest that seasonal and geographical variation of* Aedes* vectors affect the infestation in those containers in human inhabitations and surroundings.

## 1. Introduction

Dengue vectors responsible for transmission of any dengue virus serotype (DENV 1 to 4) can infest or reinfest geographically widespread areas of human inhabitations around the globe [1–5], and their breeding characteristics are diverse [4, 6, 7]. The dynamics of dengue vector ecology is constrained seasonally and geographically by ecological relationships in nature. In Southeast Asia including Thailand, two common anthropophagic dengue vectors, namely,* Aedes* (*Stegomyia*)* aegypti* (Linnaeus) and* Aedes albopictus* (Skuse), are adapted well to local environments, although the environments favorable to their infestation or reinfestation relate breeding characteristics to human settlements and activities [6–13]. As for the vectorial capacity,* Ae. aegypti* rather than* Ae. albopictus* has been responsible for transmission of any DENV serotype in urban settings rather than in rural settings.* Ae. aegypti* serves as primary dengue vector and plays significant role in vertical and transovarial dengue transmission of urban cycle [14–16].

Dengue risk estimates that the projections are strongly associated with climatic variables (temperature, precipitation, and humidity) and socioeconomic factors (population size, population density, and gross domestic product or GDP per capita) and have been shown in a warmer world for the spread of dengue in certain transmission areas or prone areas due to complex interactions of climate changes—whether globally, regionally, or locally—and socioeconomic development [17–20]. Also, the estimated risks of increase in dengue incidence are driven geographically and seasonally by increasing the proportions of the population in the urban and rural areas that have access to domestic water supply systems whether the availability of piped water or water storage tanks [17, 18]. However, most dengue risk estimates rely on nonlinearity of seasonal and geographical distributions of dengue incidence rather than dengue vectors adapted to local environments or present in receptive areas.

In Thailand, dengue vector control focuses mainly on containment of* Aedes* breeding places in the impoverished human inhabitations at which water-holding containers whether artificial or natural are abandoned in buildings and public spaces [21]. But common obstacles have been the results from the improper directions of the dengue vector surveillance and control to specific target populations and the lack of effective and sustained environmental management at household and community levels [22–24]. Regarding this, any surveyed households or containers infested with* Ae. aegypti* are always used as the units for dengue vector surveillance whether because the degree of the infestation (house index, HI or container index, and CI) is demarcated by the stratification of dengue transmission risk or the reduction of the infestation level is achieved by dengue vector control.

Moreover, the complex interactions of driving factors such as agricultural land use changes, unplanned urbanization, and physical environmental conditions [14, 18, 20, 25, 26] are considered the linkages of geographical distributions of* Aedes* vectors that can infest or reinfest in receptive areas. This infers the degree to which dengue transmission risk occurs in certain transmission areas or especially in prone areas. For instance, in the 2000s, several reports demonstrated that dengue transmission risks in urban areas of Thailand have become increasingly evident [8, 15, 23–26], as the urban-rural gradient pertaining to seasonal and geographical distribution of* Aedes* infestation remains to be established. To address this issue, the study therefore focused on land use/land cover changes; that is, land areas geographically defined in landscape structure in which human activities such as socioeconomic development, settlements/resettlements, and outdoor activities can induce changes in land use types such as urban and built-up land and agricultural land. This conceptual novelty makes the constructive urban-rural division by making use of geographical information systems- (GIS-) based land use map.

The study objective was to determine seasonal and geographical variation of dengue vectors that infested in water-holding containers in different settings of Narathiwat, Southern Thailand, where the urban-rural gradient discriminates between Muang Narathiwat district (as urban setting) and neighbor districts (as rural setting). The abundance and distribution of* Aedes* vectors that infested in different containers, as well as in households of the urban and rural settings, were compared.

## 2. Materials and Methods

### 2.1. Study Area and Design

**Figure 1:**
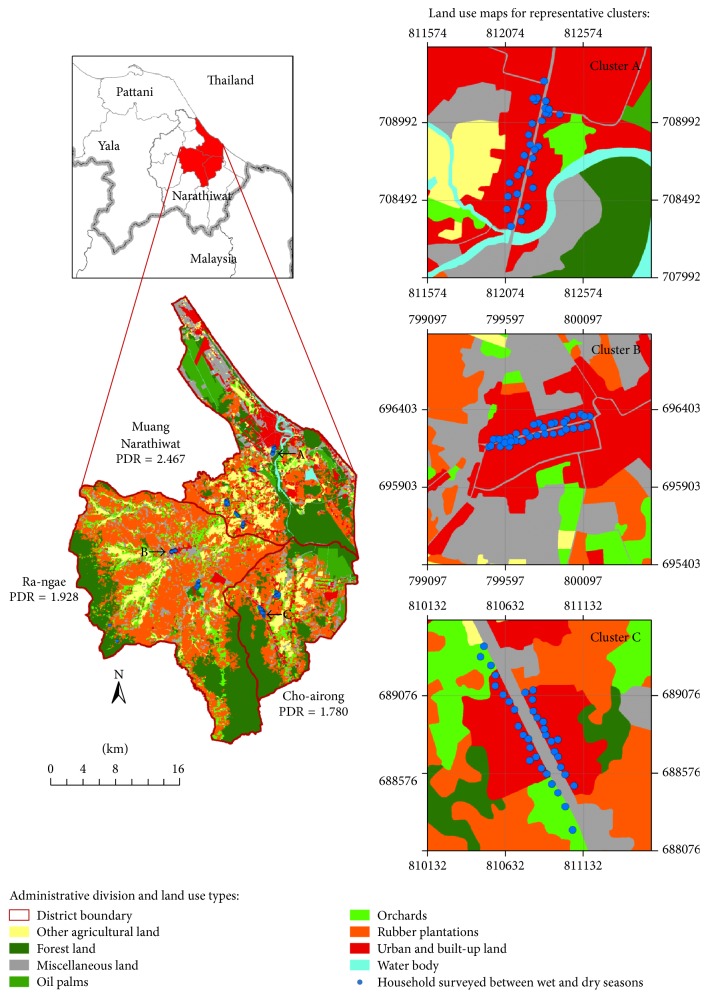
GIS-based land use map illustrated with two related urban and rural settings of Narathiwat. The relative population density ratio (PDR) for each district is shown to discriminate the urban-rural gradient. This approach was consistent with the degree of urbanization [9]. All 300 georeferenced houses that were used to conduct* Aedes* larval surveys were derived from 5 clusters of Muang Narathiwat (30 houses each), 2 clusters of Ra-ngae (40 and 35 houses), and 2 clusters of Cho-airong (40 and 35 houses). Representative cluster for each district that was assigned to a grid of 500 m^2^ is shown. Miscellaneous land included roads, landfills, pits (soil, sand, and laterite), scrubs, bamboos, marsh, swamp, grass, and others. All the land use maps that were also validated by the ground surveys between 2012 and 2013 were constructed using the ArcGIS ver 10.1 software applications.

The study area that was confined to Narathiwat province, Southern Thailand, covered three districts, namely, Muang Narathiwat, Ra-ngae, and Cho-airong (Figure 1). Based on the urban-rural division, the heterogeneity of* Aedes*-infested land area is influenced by the pattern and extent of urban and built-up land area whether high or low density of the population. There might exist diverse breeding characteristics by two different urban and rural climates of human inhabitations connecting to vegetation such as orchards mixed or not mixed with other perennial trees such as rubber trees and oil palms [6, 8, 15, 25, 26]. Thus, the relative ratio of population density adjusted to the relatively* Aedes*-infested land area of the selected district was used in this study.

With respect to the administrative level of district, the population number is a numerator and the total land area or the relatively* Aedes*-infested land area is a denominator. The relative population density ratio (PDR) for a given district is mathematically expressed as the population density adjusted to the relatively* Aedes*-infested land area (population number/the urban and built-up land area connecting to vegetation in km^2^) divided by the population density (population number/total land area in km^2^). Muang Narathiwat had a population of 115,847 (of 2012 midyear), total land area of 299.218 km^2^, and* Aedes*-infested land area of 121.293 km^2^. Ra-ngae had a population of 76,817, total land area of 435.581 km^2^, and* Aedes*-infested land area of 225.893 km^2^. Cho-airong had a population of 37,790, total land area of 185.07 km^2^, and* Aedes*-infested land area of 103.941 km^2^. Hence, we obtained the PDR for Muang Narathiwat district (PDR = 2.467) and two neighbor districts: Cho-airong (PDR = 1.780) and Ra-ngae (PDR = 1.928) (Figure 1).

The PDR value for each district was considered as the parameter that infers difference in the spatial distributions of* Aedes* vectors between the urban and rural settings. Only the season variation was considered as temporal distribution of* Aedes* infestation. This permitted a stratified cluster random sampling of total 300 houses (150 houses for each study setting); all of which were georeferenced and monitored to determine whether any household and any water-holding containers were infested with* Ae. aegypti* or* Ae. albopictus*, between wet and dry seasons. Both households and containers were used as the unit of analysis throughout the study.

### 2.2. *Aedes* Larval Survey and Monitoring

**Figure 2:**
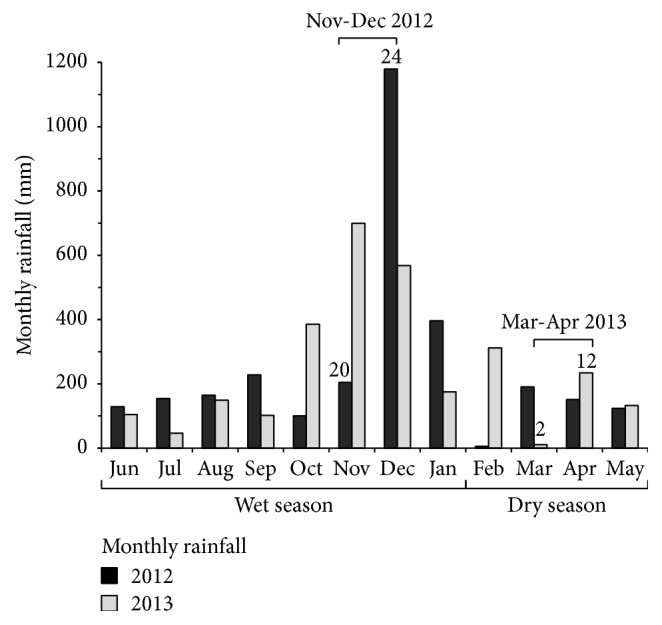
Monthly rainfall data for a period of two consecutive years, 2012-2013. Difference in the monthly rainfall (millimeters) was observed during which the* Aedes* larval surveys were conducted between wet season (November-December 2012) and dry season (March-April 2013). Also, the numberings inside and outside the bars indicate rainfall days that were observed during the studied months of wet season, November (20) and December (24), and of dry season, March (2) and April (12), respectively. These meteorological data were obtained from the Narathiwat Meteorological Station.

As mentioned above, the entomological surveys were conducted between the wet (November-December 2012) and dry (March-April 2013) seasons, based on the baseline meteorological data such as monthly rainfall and rainfall days (Figure 2). Mean monthly rainfall and rainfall days in 2012–2014 wet season are 687.15 mm and 21 days, whereas, in 2012–2014 dry season, there were 123.7 mm and 7.5 days. Prior to the* Aedes* larval survey in 2012, two entomological survey teams including the authors were trained in how to standardize qualitatively the cluster random sampling of houses as well as in how to observe, collect any larva or pupa found in any water-holding containers, and record the information of any water-holding container type, whether artificial or natural, found in any household as described below. Because environmental cleaning practices at household level [15, 21–23] might influence the indoor and outdoor distributions of water-holding containers whether or not they yielded larva productivity in any season, the surveyed containers that did not contain water were not recorded. All the surveyed households were georeferenced, and photographic evidence was recorded to compare whether the indoor and outdoor distributions of water-holding containers found between two seasons were the same. In any positive water-holding container, any larva or pupa stage of* Ae. aegypti* or* Ae. albopictus* initially monitored using the flashlight was collected, placed into a 200 mL plastic cup with cover [9], and then transferred to the laboratory. Then, pictorial keys for the identification of* Ae. aegypti* or* Ae. albopictus* [27] were used to taxonomically examine any larva found in any positive container by the entomological experts.

### 2.3. Water-Holding Container-Level Information

Regarded as breeding places for* Ae. aegypti* and* Ae. albopictus*, both artificial and natural water-holding containers were monitored, during the day time, inside and outside the houses as mentioned earlier. The indoor location was the place at which any water-holding container was found inside the house. The outdoor location was the place at which any water-holding container was found under the roof or within a radius of 5–10 meters of the premise [6]. The artificial water-holding containers were initially recorded as types and materials. Then they were categorized into 3 types of containers: water storage containers including small to large earthen jars, cement tanks, plastic drums, or bath basins; discarded receptacles including used tyres, bottles, cans, ice bins, buckets, boxes, boats, or cars; miscellaneous containers including flowerpots, flower vessels, saucers, refrigerator drain pans, cabinet ant traps, or cup/bowl/bottle water feeders [21]. Other natural containers were also recorded as types such as coconut shells, bamboo stumps, tree holes, and leaf axils [21]. Larva or pupa collection from any water-holding container was done between 08 00 and 17 00 h as described earlier.

Regardless of the kind of* Aedes* vectors, the container index (CI) was used to describe seasonal distributions of the* Aedes* infestation in container types between urban and rural settings. The CI value that infers the* Aedes* larva/pupa-producing container found in any surveyed household in any season was derived by calculating the number of any positive containers in total of surveyed water-holding containers, multiplied by 100.

### 2.4. Household-Level Information

During* Aedes* larval survey in the urban or rural setting, any infested household was defined as having a water-holding container that contained at least one larva or pupa stage of* Ae. aegypti* and/or* Ae. albopictus*. The number of* Aedes*-infested households was further analyzed temporally and spatially for distributions of* Aedes* vectors between two study settings. The house index (HI, %) was used to describe seasonal distributions of the* Aedes* infestation in receptive households between urban and rural settings. The HI value infers the number of receptive households that produce any positive containers with any* Aedes* vectors seasonally in the study setting. The HI (%) was derived by calculating the number of positive households, whether infested with* Ae. aegypti, Ae. albopictus*, or both in total of surveyed households, multiplied by 100.

### 2.5. Seasonal and Geographical Analysis of* Aedes* Infestation

Seasonal and geographical analysis of* Aedes* vectors that infested in different container types was based on the assumption of binomial distribution of the* Aedes* vectors that infested in two related urban and rural settings of Narathiwat; that is, any larvae of* Ae. aegypti* or* Ae. albopictus*, or both were found in any water-holding containers inside or outside the houses between wet and dry seasons. In this study, none or a very small number of the pupae found in all the containers were negligible. When using the container as the unit of analysis, McNemar's test was used to compare the proportions or test the difference between the proportions with two-sided *P* value < 0.05, by performing on 2 × 2 contingency table with two discrete dichotomous variables. The null hypothesis of marginal homogeneity is an equal distribution of water-holding containers, as well as* Aedes*-infested ones, between urban and rural settings whether they are distributed seasonally and domestically. As for the abundance of* Aedes* vectors, mean (±2 standard errors or SE) was presented to describe the larva numbers of* Ae. aegypti* or* Ae. albopictus* in containers observed between two seasons in the urban or rural settings of Narathiwat. The Mann-Whitney *U* test (*P* < 0.05) was used to determine whether larva numbers of* Ae. aegypti* or* Ae. albopictus* found in each type of containers in wet season are lower or higher than that in the other dry season by comparing the mean ranks of each distribution of* Aedes* larvae. The null hypothesis is that the distributions are identical and the mean ranks are the same for two-independent samples.

When using the household as the unit of analysis, McNemar's test was used as before to compare the proportions of the cluster sample of households in the urban setting (*n* = 150) or rural setting (*n* = 150) that were infested with* Aedes* vectors between two seasons. Because both urban and rural settings seemed to have similar distributions of* Aedes*-infested households, mean percentages (±2 SE) of CI values for two-independent samples were presented. The mean CI (%) that infers the distribution of positive containers in individual household infested with* Aedes* vectors was derived by calculating the percentage or proportion of positive containers in a total of water-holding containers found in an infested household in any season. The Mann-Whitney *U* test (*P* < 0.05) was used as before to test the equal distribution of mean CI values independently observed between two seasons in two study settings.

## 3. Results

### 3.1. Water-Holding Container as the Unit of Analysis

A total of 4,441 water-holding containers observed in two seasons included 2,193 (49.4%) containers from the urban setting and 2,248 (50.6%) containers from the rural setting (Table 1). On the other hand, artificial containers (94%) served as key containers, fifteenfold higher than natural containers (6%), as shown in Table 1. However, it was likely to show temporal and spatial distributions of water-holding containers whether artificial or natural. As for artificial container type, there was significant difference in the outdoor and indoor distributions of water-holding containers that were independently observed only in wet season between the urban and rural settings (*P* = 0.002). As for natural container type, there was significant difference in the outdoor and indoor distributions of water-holding containers that were independently observed in wet season between the urban and rural settings (*P* < 0.001). Meanwhile, all natural containers were found only indoors in dry season.

When analyzed for* Aedes* infestation as a result of the urban-rural division, Table 2 shows temporal and spatial distributions of* Aedes* vectors that infested in 4 container types regardless of the kind of* Aedes* vectors. Regardless of container type, the* Aedes* infestation level of 18.8% (835/4,441), CI: 9.6% (425/4,441) for wet season, and 9.2% (410/4,441) for dry season was observed. The urban and rural settings differed significantly in the outdoor and indoor distributions of* Aedes*-infested water storage containers that were independently observed in both wet (*P* < 0.001) and dry (*P* < 0.001) seasons. Similarly, the urban and rural settings differed significantly in the outdoor and indoor distributions of* Aedes*-infested miscellaneous containers (*P* = 0.003), as well as* Aedes*-infested natural containers (*P* < 0.001); all were independently observed only in wet season. As for* Aedes*-infested discarded receptacle type, none was found indoors between two seasons in both study settings.

**Figure 3:**
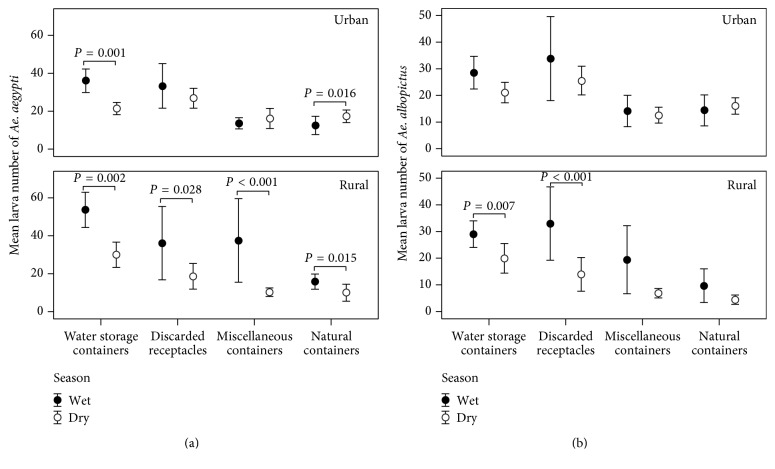
Comparisons of the abundance of* Ae. aegypti* and* Ae. albopictus* in each container type by season. The abundance of* Aedes* vectors was presented by mean larva numbers (±2 SE) of* Ae. aegypti* (a) and* Ae. albopictus* (b) as denoted by error bars. Only the outdoor distributions of* Aedes* larva numbers in any key containers were further analyzed by the independent observations between two seasons in two study settings. In the urban-rural gradient, the equal distributions of* Aedes* larvae independently observed between seasons were tested using the Mann-Whitney *U* test; the only two-independent samples that provided statistically significance (*P* < 0.05) are shown.

Based on these independent observations of* Aedes* vectors that infested in the water-holding containers but likely bred outdoors rather than indoors (Table 2), we omitted the likelihood that larval abundances of* Aedes* vectors were influenced by indoor breeding characteristics as the result of the urban-rural division in this empirical analysis (Figure 3). Because the pupa number found in any containers was negligible, only the abundance of* Aedes* vectors, that is, presented by mean larva numbers (±2 SE) of* Ae. aegypti* (Figure 3(a)) and* Ae. albopictus* (Figure 3(b)), was further analyzed by comparing the abundance of* Ae. aegypti* and* Ae. albopictus* in any outdoor containers of each container type that produced larvae between two seasons in two urban and rural settings.

In Figure 3(a), the urban setting elicited seasonal distributions (wet versus dry) of the* Ae. aegypti*-infested water storage containers (*n* = 85 versus 80), discarded receptacles (*n* = 22 versus 64), miscellaneous containers (*n* = 38 versus 45), and natural containers (*n* = 20 versus 26). Seasonal distributions of* Ae. aegypti* larvae observed only in the water storage containers and natural containers were remarkably noticed. The distributions of* Ae. aegypti* larva numbers in water storage containers observed in dry season were significantly lower than that observed in other wet seasons (*Z* = −3.252, *P* = 0.001). The distributions of* Ae. aegypti* larva numbers in natural containers observed in dry season were significantly lower than that observed in other wet seasons (*Z* = −2.410, *P* = 0.016). Similar to the urban setting, the rural setting showed seasonal distributions (wet versus dry) of* Ae. aegypti-*infested water storage containers (*n* = 97 versus 61), discarded receptacles (*n* = 15 versus 57), miscellaneous containers (*n* = 118 versus 49), and natural containers (*n* = 15 versus 17) (Figure 3(a)). However, the rural settings contrasted seasonal distributions of* Ae. aegypti* larvae in all 4 container types. The distributions of* Ae. aegypti* larvae observed in dry season were significantly lower than that observed in other wet seasons: water storage containers (*Z* = −3.040, *P* = 0.002), discarded receptacles (*Z* = −2.195, *P* = 0.028), miscellaneous containers (*Z* = −6.185, *P* < 0.001), and natural containers (*Z* = −2.425, *P* = 0.015).

In Figure 3(b), the urban setting also elicited seasonal distributions (wet versus dry) of the* Ae. albopictus*-infested water storage containers (*n* = 84 versus 78), discarded receptacles (*n* = 22 versus 60), miscellaneous containers (*n* = 36 versus 46), and natural containers (*n* = 19 versus 27). Unlike that of* Ae. aegypti*-infested containers, the seasonal distributions of* Ae. albopictus* larvae in any containers were not pronounced. Similar to the urban setting, the rural setting also exhibited seasonal distributions (wet versus dry) of* Ae. albopictus-*infested water storage containers (*n* = 102 versus 58), discarded receptacles (*n* = 17 versus 52), miscellaneous containers (*n* = 101 versus 42), and natural containers (*n* = 14 versus 15) (Figure 3(b)). Only seasonal distributions of* Ae. albopictus* larvae in the water storage containers and discarded receptacles were remarkably noticed. The distributions of* Ae. albopictus* larvae observed in dry season were significantly lower than that observed in wet season: water storage containers (*Z* = −2.677, *P* = 0.007) and discarded receptacles (*Z* = −4.077, *P* < 0.001).

### 3.2. Household as the Unit of Analysis

**Figure 4:**
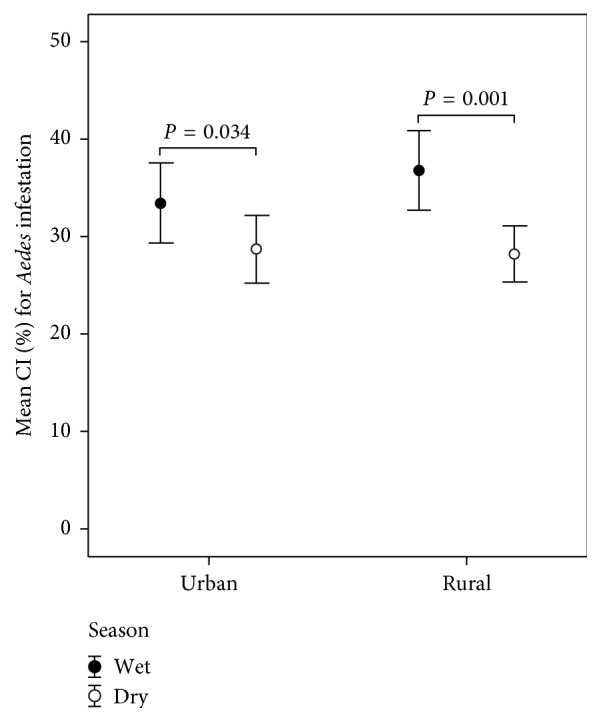
Comparisons of the* Aedes*-producing containers in the infested households of the urban and rural settings by season. Mean percentages (±2 SE) of CI values as denoted by error bars were presented between two study settings. The statistical significance with the Mann-Whitney *U* test (*P* < 0.05) is shown for two categorical seasons on the distributions of* Aedes* larva numbers.

Table 3 shows the temporal and spatial distributions of* Aedes* vectors that infested households in the urban (*n* = 150) and rural (*n* = 150) settings, regardless of the kind of* Aedes* vectors. There was significant difference in the proportions of households infested with* Aedes* vectors between the urban and rural settings (*P* = 0.022) when analyzed for only the independent observation by dry season. When the* Aedes* infestation was compared between the urban and rural climates, only the* Aedes*-infested households that have* Aedes* larva-producing containers were considered (Figure 4). The urban setting (*n* = 150) included 80 (53.3%) and 94 (62.7%)* Aedes-*infested households observed between wet and dry seasons. The rural setting (*n* = 150) included 86 (57.3%) and 84 (56.0%)* Aedes-*infested households independently observed between wet and dry seasons. On the other hand, the distributions of mean CI values in dry season were significantly lower than that in other wet seasons; for the urban setting, *Z* = −2.125, *P* = 0.034; for the rural setting, *Z* = −3.246, *P* = 0.001 (Figure 4).

## 4. Discussion

Like other receptive areas of Southeast Asia including Thailand [6, 8, 9], Philippines [7], and Malaysia [13, 28], Narathiwat bordered by Malaysia has experienced the land use and land cover change, although linked with diverse spatiotemporal distributions of* Aedes* vectors. The spatiotemporal distribution of dengue vectors like* Ae. aegypti and Ae. albopictus* that can infest or reinfest artificial and natural water-holding containers in the impoverished human inhabitations is often indirect and dynamic over space and time. In present study, it was clear to note that the artificial containers served as key water-holding containers available for* Aedes* breeding in the urban and rural settings. During wet season, the rural setting produced more artificial containers available for indoor rather than outdoor breeding of* Aedes* vectors. During dry season, there was tendency of the availability of more artificial containers in both urban and rural settings, but the outdoor and indoor distributions were similar. As for the natural container type, there was tendency of more natural containers available for outdoor breeding of* Aedes* vectors. To explore what breeding characteristics of* Aedes* vectors are, it was clear that, as seen in Table 2 and Figure 3,* Aedes* vectors produced the offspring by breeding outdoors rather than indoors in all 4 container types available during wet season rather than during dry season. Hence, spatiotemporal distributions of more artificial containers might contribute substantially to the greater variability of larval abundances (per container type) and container index (per house of each study setting) between urban and rural settings.

Water storage containers (especially jars, tanks, bath basins, and drums) [6–8, 12, 28] were more likely to be favorable for* Aedes* vectors to breed outdoors in both wet and dry seasons. However, the spatiotemporal distributions of* Aedes* vectors that infested in water storage containers were not proportional to size, even though their indoor and outdoor distributions were similar for both seasons (data not shown). It was not surprising that the people residing in rural rather than urban setting—whether or not they had access to piped water—stored rainwater or other water sources for domestic use in as many as jars, tanks, bath basins, or drums during wet season. This is because there is tendency of shortage of water supply during dry season as seen in Figure 2. Moreover, many* Aedes*-infested water storage containers found in this study were laid outdoors or under the roof and were not covered with any cover types. In wet season, rural setting yielded outdoors versus indoors larval abundances, 21.4 versus 3.6* Ae. aegypti* larvae and 12.2 versus 2.0* Ae. albopictus* larvae per water storage container. Urban setting yielded outdoors versus indoors larval abundances, 14.4 versus 0.6* Ae. aegypti* larvae and 11.6 versus 0.1* Ae. albopictus* larvae per water storage container. In dry season, rural setting yielded outdoors versus indoors larval abundances, 9.2 versus 0.9* Ae. aegypti* larvae and 5.2 versus 0.9* Ae. albopictus* larvae per water storage container. Urban setting yielded outdoors versus indoors larval abundances, 4.8 versus 0.9* Ae. aegypti* larvae and 4.8 versus 0.5* Ae. albopictus* larvae per water storage container.

Miscellaneous containers (especially saucers, cabinet ant traps, and flowerpots) [6–8] were also favorable to breed* Aedes* vectors indoors rather than outdoors. Unlike water storage container type, the miscellaneous containers infested with* Aedes* vectors were proportional to size for both seasons and study settings (data not shown). This was because many households from both urban and rural settings produced household decorating items or accessories that were abandoned indoors rather than outdoors and often became water-holding containers that produced larval abundances. However, when examined for larval abundances, the outdoor breeding for* Aedes* vectors showed the densities greater than the indoor breeding. In wet season, rural setting yielded outdoors versus indoors larval abundances, 21.8 versus 3.1* Ae. aegypti* larvae and 10.2 versus 1.1* Ae. albopictus* larvae per miscellaneous container. Urban setting yielded outdoors versus indoors larval abundances, 3.5 versus 1.0* Ae. aegypti* larvae and 4.3 versus 0.7* Ae. albopictus* larvae per miscellaneous container. In dry season, urban setting yielded outdoors versus indoors larval abundances, 6.2 versus 1.2* Ae. aegypti* larvae and 4.4 versus 1.1* Ae. albopictus* larvae per miscellaneous container. Rural setting also yielded outdoors versus indoors larval abundances, 4.1 versus 0.6* Ae. aegypti* larvae and 2.2 versus 0.3* Ae. albopictus* larvae per miscellaneous container.

Discarded receptacles (especially used tyres, buckets, and ice bins) [6–8, 11, 29] were likely to be favorable for* Aedes* vectors to breed outdoors. Many households from both urban and rural settings produced household wastes that were abandoned outdoors and often became water-holding containers as potential breeding places for* Aedes* vectors. In wet season, rural setting yielded outdoors versus indoors larval abundances, 10.0 versus 3.6* Ae. aegypti* larvae and 10.4 versus 2.0* Ae. albopictus* larvae per discarded receptacle. Urban setting yielded only outdoors larval abundances, 6.6* Ae. aegypti* larvae and 6.7* Ae. albopictus* larvae per discard receptacle. In dry season, urban setting yielded only outdoors larval abundances, 6.8* Ae. aegypti* larvae and 6.0* Ae. albopictus* larvae per discarded receptacle, whereas, in rural setting, outdoors larval abundances were 4.7* Ae. aegypti* larvae and 3.2* Ae. albopictus* larvae per discarded receptacle.

Natural containers (especially coconut shells) [6–8, 28] were also preferred outdoor breeding places for* Aedes* vectors. In wet season, rural setting yielded outdoors versus indoors larval abundances, 5.4 versus 10.0* Ae. aegypti* larvae and 3.3 versus zero* Ae. albopictus* larvae per natural container. Urban setting yielded only outdoors larval abundances, 5.4* Ae. aegypti* larvae and 5.9* Ae. albopictus* larvae per natural container. In dry season, urban setting yielded only outdoors larval abundances, 3.6* Ae. aegypti* larvae and 3.4* Ae. albopictus* larvae per natural container, whereas, in rural setting, outdoors larval abundances were 2.9* Ae. aegypti* larvae and 1.1* Ae. albopictus* larvae per natural container.

Among the 4 container types, the water storage containers used by the households in the rural settings of Narathiwat were likely to show the preponderance of larval habitats of* Aedes* vectors in both wet and dry seasons. However, the outdoor breeding for* Aedes* vectors in all 4 container types was likely to produce larval abundances higher than the indoor breeding. When analyzed for the receptive setting that* Aedes* vectors can infest temporally, our findings demonstrated that both urban and rural settings seemed to have* Aedes* larva-producing containers in wet season rather than in dry season, as seen in Figure 4. When analyzed for the* Aedes* larval abundance, such the findings agreed with the rainfall data that* Aedes* larval abundances were related to monthly rainfall. The findings implied that there was greater availability of breeding places for* Aedes* vectors in wet season than in dry season. The seasonal variation was likely to regulate the abundance and distribution of* Aedes* vectors in Narathiwat. But there was greater variability of larval abundances of* Ae. aegypti* and* Ae. albopictus* in different container types due to the factors underlying water storage behaviors and garbage management. In other words, the aspect of the urban-rural division divided by PDR values at the district level might influence the delineation of urban (high degree of heterogeneous landscape) and rural (low degree of heterogeneous landscape) settings upon the cluster sampling. The urban-rural division of the studied districts may be influenced by the PDR threshold values among the cluster samples. House characteristics might become the underlying factor that influences* Aedes* larva-producing containers, but there seemed to be indistinguishable from each other in this study. This may be a reason why two study settings (urban versus rural) were likely to show the stability of* Aedes* infestation levels: 17.8% versus 21.1% CI per house in wet season and 18.0% versus 15.8% CI per house in dry season.

In addition, the effective and sustained dengue vector control in Thailand has focused radically on the household-level practices in environmental cleaning (PEC) practices through dengue prevention and control campaign as the part of the National Dengue Prevention and Control Program (NDCP). In each province implementing the NDCP, if expected to accompany the campaign activity and the adoption of other appropriately designed dengue vector control measures, the enhancement of the household-level PEC practices has been thought to reach the achievable targets at both village and subdistrict levels. The dengue prevention and control campaign is often operated before, during, or after the dengue outbreaks in order to ascertain that the conveyed messages can penetrate most households by making use of all media and channels. Nonetheless, there were no strict directions on whether all the implementing provinces determine the degree to which any households operate PEC practices routinely despite the fact that PEC practices guided by the levels of central, provincial, and local health sectors are expected to have the consequences of reducing the desired level of* Aedes* infestation (CI and HI).

Evidently, this study provided the proof that the household-level PEC practiced by the households from both urban and rural settings of Narathiwat had the impacts on larval abundances of* Ae. aegypti* in 3 artificial container types and hence the containment of* Aedes* breeding places. For instance, the intermittent household-level PEC practices for both urban and rural settings contributed strongly to the significant reduction of outdoor larval abundances of* Ae. aegypti* that was remarkably shown in Figure 3 for water storage containers as well as miscellaneous containers. However, discarded receptacle was only the container type that the people paid no or less attention to perform household-level PEC practices. Although discarded receptacles yielded larva productivity lower than water storage container and miscellaneous container, there is however a need for dengue vector surveillance and control to address them as potential breeding sites, as well as to diminish or dispose them in timeliness manner [21, 24]. The findings might imply that, if expected to reach the conveyed messages of household-level PEC practices, most households from both urban and rural settings played significant role in the containment of* Aedes* breeding places. However, the study could not provide the reason why some households neglected PEC practices because there exist as many as key containers that still had larval abundances and were not cleaned.

In summary, the seasonal and geographical variations of* Aedes* vectors were dynamic in nature. Less urbanization is also important for dengue transmission risk. As the result of the urban-rural gradient, the rural ecology of dengue also relates the seasonal distributions of* Aedes* vectors to dengue transmission dynamics. This is because there was greater availability of more container types as potential breeding sites, especially for* Ae. aegypti* that had more competitive advantage than* Ae. albopictus* and contributed greatly to produce larval abundances in spatial overlap. The study suggests that the higher urban-rural gradient, the greater the risk for seasonal and geographical distributions of* Aedes* vectors that persistently infest in 4 container types as potential breeding sites in human inhabitations and surroundings. To mitigate the dengue transmission risks, the effective and sustained household-level PEC practices should be applied to all receptive areas to reduce the intensity of larval abundances and hence contain* Aedes* breeding places at both village and subdistrict levels. Furthermore, like other receptive areas of Malaysia [13, 28, 30], Narathiwat experienced cobreeding of* Ae. aegypti* and* Ae. albopictus* in both urban and rural settings as this epidemiological implication for dengue/chikungunya virus transmission needs further investigation. Such anthropogenic land use and land cover change that contributes greatly to the health impacts of vector-borne diseases such as malaria [31] and dengue [32] also needs to be logically analyzed to determine the seasonal and geographical distributions of* Aedes* vectors.

## Figures and Tables

**Table 1:** Seasonal and geographical distributions of water-holding containers.

Container type	Season	Location	Number (%) of containers surveyed between the study settings			*P* value
			Urban	Rural	Total	
Artificial	Wet	Outdoor	383	404	787 (41.1)	0.002^*∗*^
		Indoor	496	633	1,129 (58.9)	
		Total	879 (45.9)	1,037 (54.1)	1,916	
	Dry	Outdoor	629	470	1,099 (48.8)	0.180
		Indoor	513	640	1,153 (51.2)	
		Total	1,142 (50.7)	1,110 (49.3)	2,252	

Natural	Wet	Outdoor	46	40	86 (97.7)	<0.001^*∗*^
		Indoor	0	2	2 (2.3)	
		Total	46 (68.1)	42 (47.9)	88	
	Dry	Outdoor	126	59	185 (100)	NA
		Indoor	0	0	0	
		Total	126 (68.1)	59 (31.9)	185	

NA: not applicable.

^*∗*^Statistically significant with McNemar's test for two-independent samples.

**Table 2:** Seasonal and geographical distributions of *Aedes* vectors in water-holding containers.

Container type	Season	Location	Number (%) of positive containers surveyed between the study settings			*P* value
			Urban	Rural	Total	
Water storage	Wet	Outdoor	84	81	165 (86.4)	<0.001^*∗*^
		Indoor	4	22	26 (13.6)	
		Total	88 (46.1)	103 (53.9)	191	
	Dry	Outdoor	76	54	130 (89.7)	<0.001^*∗*^
		Indoor	8	7	15 (10.3)	
		Total	84 (57.9)	61 (42.1)	145	

Discarded receptacles	Wet	Outdoor	22	17	39 (100)	NA
		Indoor	0	0	0	
		Total	22 (56.4)	17 (43.6)	39	
	Dry	Outdoor	65	57	122 (100)	NA
		Indoor	0	0	0	
		Total	65 (53.3)	57 (46.7)	122	

Miscellaneous containers	Wet	Outdoor	15	52	67 (41.9)	0.003^*∗*^
		Indoor	25	68	93 (58.1)	
		Total	40 (25.0)	120 (75.0)	160	
	Dry	Outdoor	11	26	37 (37.4)	0.162
		Indoor	38	24	62 (62.6)	
		Total	49 (49.5)	50 (50.5)	99	

Natural containers	Wet	Outdoor	20	14	34 (97.1)	<0.001^*∗*^
		Indoor	0	1	1 (2.9)	
		Total	20 (57.1)	15 (42.9)	35	
	Dry	Outdoor	27	17	44 (100)	NA
		Indoor	0	0	0	
		Total	27 (61.4)	17 (38.4)	44	

NA: not applicable.

^*∗*^Statistically significant with McNemar's test for two-independent samples.

**Table 3:** Seasonal and geographical distributions of *Aedes* vectors at household level.

Season	Households infested with *Aedes* vectors	Number (%) of households surveyed between the study settings			*P* value
		Urban	Rural	Total	
Wet	Yes	80	86	166 (55.3)	0.238
	No	70	64	134 (44.7)	
	Total	150 (50.0)	150 (50.0)	300	

Dry	Yes	94	84	178 (59.3)	0.022^*∗*^
	No	56	66	122 (40.7)	
	Total	150 (50.0)	150 (50.0)	300	

^*∗*^Statistically significant with McNemar's test for two-independent samples.
